# Positive end-expiratory pressure induced changes in airway driving pressure in mechanically ventilated COVID-19 Acute Respiratory Distress Syndrome patients

**DOI:** 10.1186/s13054-023-04345-5

**Published:** 2023-03-21

**Authors:** Mônica Rodrigues da Cruz, Luciana Moisés Camilo, Tiago Batista da Costa Xavier, Gabriel Casulari da Motta Ribeiro, Denise Machado Medeiros, Luís Felipe da Fonseca Reis, Bruno Leonardo da Silva Guimarães, André Miguel Japiassú, Alysson Roncally Silva Carvalho

**Affiliations:** 1 Instituto Nacional de Infectologia Evandro Chagas, Fundação Oswaldo Cruz (INI/Fiocruz), Rio de Janeiro, Brasil; 2 Hospital Universitário Pedro Ernesto, Universidade do Estado do Rio de Janeiro (HUPE/UERJ), Rio de Janeiro, Brasil; 3 Instituto de Educação, Ciência e Tecnologia do Rio de Janeiro (IFRJ), Rio de Janeiro, Brasil; 4 Programa Doutor Empreendedor, Fundação Carlos Chagas Filho de Amparo à Pesquisa do Estado do Rio de Janeiro, Rio de Janeiro, Brasil; 5 Hospital Central da Polícia Militar (HCPM), Rio de Janeiro, Brasil; 6 Hospital Niterói D’Or, Rio de Janeiro, Brasil; 7 Laboratório de Fisiologia da Respiração, Instituto de Biofísica Carlos Chagas Filho, Universidade Federal do Rio de Janeiro (IBCCF/UFRJ), Rio de Janeiro, Brasil; 8 Instituto D’or de Pesquisa e Ensino, Rio de Janeiro, Brasil; 9 Hospital Barra D’Or, Rio de Janeiro, Brasil; 10 Programa de Pós-Graduação em Ciências da Reabilitação, Centro Universitário Augusto Motta (UNISUAM), Rio de Janeiro, Brasil

**Keywords:** Positive end-expiratory pressure, Acute Respiratory Distress Syndrome, Electrical impedance tomography, COVID-19, Mechanical ventilation

## Abstract

**Background:**

The profile of changes in airway driving pressure (dP_aw_) induced by positive-end expiratory pressure (PEEP) might aid for individualized protective ventilation. Our aim was to describe the dP_aw_ versus PEEP curves behavior in ARDS from COVID-19 patients.

**Methods:**

Patients admitted in three hospitals were ventilated with fraction of inspired oxygen (FiO_2_) and PEEP initially adjusted by oxygenation-based table. Thereafter, PEEP was reduced from 20 until 6 cmH_2_O while dP_aw_ was stepwise recorded and the lowest PEEP that minimized dP_aw_ (PEEPmin_dP_aw_) was assessed. Each dP_aw_ vs PEEP curve was classified as J-shaped, inverted-J-shaped, or U-shaped according to the difference between the minimum dP_aw_ and the dP_aw_ at the lowest and highest PEEP. In one hospital, hyperdistention and collapse at each PEEP were assessed by electrical impedance tomography (EIT).

**Results:**

184 patients (41 including EIT) were studied. 126 patients (68%) exhibited a J-shaped dP_aw_ vs PEEP profile (PEEPmin_dP_aw_ of 7.5 ± 1.9 cmH_2_O). 40 patients (22%) presented a U (PEEPmin_dP_aw_ of 12.2 ± 2.6 cmH_2_O) and 18 (10%) an inverted-J profile (PEEPmin_dP_aw_ of 14,6 ± 2.3 cmH_2_O). Patients with inverted-J profiles had significant higher body mass index (BMI) and lower baseline partial pressure of arterial oxygen/FiO_2_ ratio. PEEPmin_dP_aw_ was associated with lower fractions of both alveolar collapse and hyperinflation.

**Conclusions:**

A PEEP adjustment procedure based on PEEP-induced changes in dP_aw_ is feasible and may aid in individualized PEEP for protective ventilation. The PEEP required to minimize driving pressure was influenced by BMI and was low in the majority of patients.

## Introduction

Hypoxemic respiratory failure is the leading cause of intensive care unit (ICU) admission in COVID-19, the majority of subjects meeting Acute Respiratory Distress Syndrome criteria (C-ARDS) [1]. Initially, it was observed that many patients presented a disparity between well-preserved lung mechanics and severe hypoxemia [2] and 2 different phenotypes in C-ARDS were proposed, which should be managed with different ventilatory strategies [2]. However, this was not confirmed in posterior published data, remaining recommendations to treat C-ARDS accordingly ARDS ventilation evidence-based [3]. Several hypotheses were proposed to the wide range of respiratory system compliance (Crs) observed in many C-ARDS series, including hypoxemia due to impaired perfusion in patients with higher compliance or lungs with high recruitability and lower compliance [2].

Optimal positive end-expiratory pressure (PEEP) has been pursued [4] and the question of how to recognize patients that get benefit from higher PEEP levels has led to new technologies like Electrical Impedance Tomography (EIT), a bedside tool to monitor ventilation distribution, allowing PEEP titration to reduce both collapse and hyperdistention [5].

Airway driving pressure (dP_aw_) is a simple parameter to monitor on the ventilator and, when diminished with increased PEEP was associated with reduced mortality risk in ARDS [6]. In C-ARDS, a lower dP_aw_ was associated with better survival [7, 8].

In the present study, we aim to describe the profile of PEEP-induced changes in dP_aw_ during a PEEP adjustment procedure as aid for individualized protective ventilation, including a group where it was done together with an EIT monitor.

## Methods

### Patients

In this prospective observational physiologic study, adults patients admitted to the ICU of three hospitals with C-ARDS confirmed by positive nasopharyngeal polymerase chain reaction for SARS-CoV-2 and receiving invasive mechanical ventilation (MV) ≤ 48 h were analyzed. Patients with barotrauma assessed by computed tomography (CT), chronic pulmonary disease, and increased intracranial pressure were excluded.

### Mechanical ventilation settings

After analgesia and sedation adjustment, all subjects were initially ventilated in volume-controlled ventilation, tidal volume of 6 mL/kg with constant inspiratory flow, plateau pressure ≤ 30 cmH_2_O, FiO_2_ and PEEP adjusted to keep SaO_2_ > 90% based on the ARDSNetwork table [9] and respiratory rate to maintain normal partial pressure of carbon dioxide (PaCO_2_). Fluids and vasopressors were provided to maintain mean arterial pressure above 60 mmHg and, neuromuscular blocking used to avoid ventilatory asynchronies.

### PEEP adjustment procedure

After initial ventilatory settings, PEEP was reduced, 2 cmH_2_O every thirty seconds [10], from 20 until 6 cmH_2_O while dP_aw_ was assessed in each step, and the lowest PEEP that minimized dP_aw_ (PEEPmin_dP_aw_) was identified. The posterior PEEP adjustment was at the discretion of the clinical team responsible for patient care.

### EIT assessment

In one of the hospitals, patients were investigated by EIT (Enlight 1800, Timpel, São Paulo, Brazil) during the PEEP adjustment procedure. Regional variations in impedance (∆*Z*) during ventilation, map the *V*_t_ distribution in the lung and creates a PEEP titration tool which was used to assess PEEP-induced pulmonary hyperdistention and collapse and its effects on dP_aw_ during the PEEP adjustment procedure. The EIT optimal PEEP (PEEP_EIT_) was defined as the PEEP that represents the best compromise between hyperdistention and collapse estimated [5, 11].

### Evaluation of dP_aw_ vs PEEP curve profile

After the PEEP adjustment procedure, each dP_aw_ vs PEEP curve was recorded and *retrospectively* classified into one of three categories according to the difference between the minimum dP_aw_ [12] and the dP_aw_ at the lowest (ΔdPlow) and highest (ΔdPhigh) PEEP [4]. If ΔdPlow < 0.2 × ΔdPhigh, the curve was classified as J-shaped; if ΔdPhigh < 0.2 × ΔdPlow, the curve was classified as inverted-J-shaped; otherwise, the curve was U-shaped.

### Statistical analysis

Results are reported without imputation as mean (standard deviation), or count (percentage), after testing for normality using the Shapiro–Wilk test. One-way ANOVA was used for the comparison between the three groups. A Bonferroni-Holm post hoc test was applied to correct multiple testing. Hyperdistention and collapse curves at different PEEP levels were assessed by computing areas under the curves (AUCs) [13] by adding the areas under each pair of consecutive observations:

$${\text{AUC}} = \frac{1}{2}\mathop \sum \limits_{1}^{8} \left( {{\text{PEEP}}_{i + 1} - {\text{PEEP}}_{i} } \right) \times \left( {Y_{i + 1} + Y_{i} } \right),$$

where *Y* was the estimated hyperdistention or collapse. The AUCs were compared only between U-shaped and J-shaped PEEP vs dP_aw_ groups, because just one patient with Inverted-J shape had EIT measurement.

Statistical analysis was performed in R (The R Foundation, Vienna, Austria), and a *p* < 0.05 was considered significant.

## Results

Between Jul 27th, 2020, and Feb 24th, 2021, a total of 184 patients were included, and a PEEP adjustment procedure was performed before 48 h on invasive MV. Table 1 shows clinical characteristics in each curve profile dP_aw_ vs PEEP. Patients with inverted J-Shaped dP_aw_ versus PEEP profile presented significantly higher body mass index (BMI) (Table 1) and lower partial pressure of arterial oxygen and fraction of inspired oxygen ratio (PaO_2_/FiO_2_) and Crs at baseline (Table 2).

**Table 1:** Characteristics of patients with C-ARDS enrolled in the PEEP titration

Patients’ characteristics	All COVID-19	J-shaped *n* = 126	U-shaped *n* = 40	Inverted J-shaped *n* = 18	*p* value
Age, years, *n* = 184	60.04 ± 15.89	60.37 ± 15.94	59.20 ± 17.30	59.67 ± 12.68	0.915
Male, *n* (%), *n* = 184	127 (69.02%)	92 (72.44%)	25 (62.50%)	10 (55.55%)	0.160
Body mass index, kg/m^2^, *n* = 183	29.02 ± 6.43	27.48 ± 6.65^a^	30.16 ± 6.95^a,b^	35.89 ± 8.67^b^	< 0.001
Comorbidities, *n* (%), *n* = 69	44 (63.8%)	30 (65.2%)	11 (68.8%)	3 (42.9%)	0.500
Hypertension, *n* (%)	38 (55.1%)	24 (52.2%)	11 (68.8%)	3 (42.9%)	0.422
Diabetes mellitus, *n* (%)	29 (42%)	18 (39.1%)	8 (50%)	3 (42.9%)	0.807
SOFA, *n* = 143	5.43 ± 4.03	5.17 ± 3.04	5.69 ± 3.39	6.53 ± 3.06	0.262
PEEP at baseline *n* = 114	10 (10–14)	10 (10–14)^a,c^	12 (10–15.5)^a^	15 (10.5–19.5)^c^	< 0.05
FiO_2_ at baseline, *n* (%), *n* = 184	80 (60–100)	70 (60–100)^a^	100 (70–100)^a^	95 (60–100)	< 0.05
PaCO_2_ at baseline, mmhg, *n* = 75	51.6 ± 11.9	51.4 ± 11.1	54.2 ± 13.2	47.1 ± 14.4	0.410
Respiratory rate at baseline, breaths/min, *n* = 75	20 (20–25)	20 (20–25)	24 (20–24.5)	20 (20–20)	0.170
Minute ventilation at baseline, L/min, *n* = 75	8.1 ± 2.0	8.3 ± 2.0	8.1 ± 1.7	6.8 ± 1.7	0.180

Continuous variables are expressed as mean and standard deviation or median and interquartile range, according to normality distribution. A one-way ANOVA or the Kruskal–Wallis test was used for the comparison between three groups with a respective post hoc analysis. The letters a, b and c express values that are statistically different. COVID-19, coronavirus disease-19; SOFA, sequential organ failure assessment score; PEEP, positive end-expiratory pressure; dP_aw_, airway driving pressure; PaO_2_/FiO_2_, partial pressure of arterial oxygen and fraction of inspired oxygen ratio; FiO_2_, fraction of inspired oxygen; PaCO_2_, partial pressure of carbon dioxide

**Table 2:** Respiratory mechanics and EIT data

Respiratory mechanics	J-shaped *n* = 126	U-shaped *n* = 40	Inverted J-shaped *n* = 18	*p* value
Tidal volume, mL/kg of IBW (mean, sd)	6.03 ± 0.03	5.86 ± 0.92	5.97 ± 0.14	0.098
Baseline Crs, mL/cmH_2_O (mean, sd)	33.47 ± 7.25^a^	29.24 ± 8.70^a,b^	25.64 ± 8.45^b^	< 0.001
Baseline dP_aw_, cmH_2_O (mean, sd)	12.65 ± 2.66^a^	13.21 ± 3.94^a,b^	15.03 ± 3.72^b^	< 0.05
Baseline PaO_2_/FiO_2_, mmHg (mean, sd)	139.32 ± 52.67^a^	120.72 ± 57.68^a,b^	92.43 ± 40.43^b^	< 0.05
PEEPmin_dP_aw_, cmH_2_O (median, IIQ)	7.52 ± 1.9^a,c^	12.2 ± 2.64^a,b^	14.6 ± 2.38^b,c^	< 0.001

EIT assessment	*N* = 28	*N* = 12	*N* = 1	
Hyperdistention at the optimal PEEP, % (mean, sd)	1.58 ± 2.34	6.34 ± 10.22	1.3 ± 0	0.071
AUC for hyperdistention, %.cmH_2_O (mean, sd)	216.75 ± 81.44^a^	116.17 ± 77.53ª	6.3 ± 0	< 0.001
Collapse at the optimal PEEP, % (mean, sd)	13.86 ± 13.38	10.81 ± 10.38	0.0 ± 0	0.473
AUC for collapse, %.cmH_2_O (mean, sd)	96.95 ± 70.40^a^	149.42 ± 95.54^a^	418 ± 0	< 0.001
EIT optimal PEEP, cmH_2_O, (mean, sd)	9.17 ± 2.53^a^	12.96 ± 3.29ª	14.22 ± 0	< 0.001

Continuous variables are expressed as mean and standard deviation or median and interquartile range, according to normality distribution. A one-way ANOVA or the Kruskal–Wallis test was used for the comparison between three groups with a respective post hoc analysis. A t-test was used for the comparison between pairs. The letters a, b and c express values that are statistically different. IBW, ideal body weight; Crs, respiratory system compliance; dP_aw_, airway driving pressure; PaO_2_/FiO_2_, ratio of partial pressure of arterial oxygen and fraction of inspired oxygen; PEEPmin_dP_aw_, lowest PEEP that minimized dP_aw_; PEEP, positive end-expiratory pressure; EIT, electrical impedance tomography; AUC, area under the curve

### Respiratory mechanics and PEEP titration

**Fig. 1:**
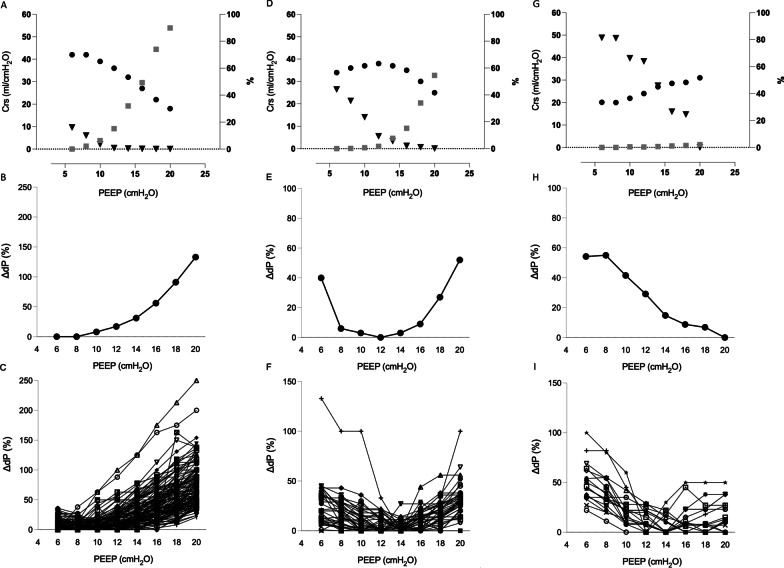
Respiratory system mechanics associated with the percentage of collapse and hyperdistention at different levels of PEEP. In panels **A**, **D**, and **G**, data were obtained by electrical impedance tomography, where ● is the respiratory system compliance; Δ is the percentage of collapse and □ is the percentage of overdistension. Panels **B**, **C**, **E**, **F**, **H**, and **I** show the percentage change in driving pressure obtained by a mechanical ventilator for a representative patient (**B**, **E**, **H**) and all patients (**C**, **F**, **I**). Panels **A–C** correspond to the category of patients with J-shaped curves; panels **D**–**F** correspond to the category of patients with U-shaped curves, and panels **G**–**I** correspond to the category of patients with inverted J-shaped curves

Based on the analysis of the dP_aw_ vs PEEP profile, most of the COVID-19 patients (*n* = 126) exhibited a J-shaped dP_aw_ vs PEEP profile with dP_aw_ starting to increase for PEEPs ≥ 7.5 ± 1.9 cmH_2_O, only a few COVID-19 patients had mostly inverted-J profiles (*n* = 18), usually requiring higher levels of PEEP (PEEPmin_dP_aw_ ranging from 14 to 20 cmH_2_O) (Table 2, Fig. 1). Only 21.7% of COVID-19 patients presented the U-shaped profile with the PEEPmin_dP_aw_ ranging from 10 to 14 cmH_2_O.

The J-shaped dP_aw_ vs PEEP profile was associated with increased hyperdistention, and collapse reduction as PEEP increased and, in this group, PEEPmin_dP_aw_ was lower than PEEP based on the ARDSNetwork table (Table 2). At the range of the PEEPmin_dP_aw_ both hyperdistention and collapse were minimized independent of the dP_aw_ vs PEEP profile (Table 2, Fig. 1).

## Discussion

Our study interpreted the dP_aw_ vs PEEP curve profile among C-ARDS patients. The main findings were: (1) 90% of C-ARDS-19 patients presented a J- or U-shaped dP_aw_ vs PEEP curve profile usually requiring PEEPs < 12 cmH_2_O to minimize dP_aw_; (2) PEEPs > 15 cmH_2_O would be necessary in only 10% of C-ARDS, and those patients presented an inverted-J dP_aw_ vs PEEP curve profile and higher BMI; and (3) PEEPmin_dP_aw_ was associated with a reduction of both alveoli collapse and hyperdistention. All these patients averaged PaO_2_/FiO_2_ below 150 which there is evidence of benefit from using higher levels of PEEP in ARDS [14].

ARDS and C-ARDS are heterogeneous conditions with uncertainty about to set PEEP [2, 3] commonly based by oxygenation targets [9]. However in C-ARDS this strategy frequently resulted in worse lung mechanics [15], and cardiac output impairment [16].

Our EIT data and an experimental CT study [4] show that, at constant *V*_T_, dP_aw_ and compliance respond to both hyperdistention and collapse. 126/184 of our patients presented a J-shaped curves, with the largest hyperdistention AUC, where increasing PEEP to improve oxygenation may not work. In U-shaped curves the balanced risk of collapse and hyperdistention was obtained with about 12 cmH_2_O PEEP. In these two groups, higher PEEPs would carry a greater risk of iatrogenesis. Finally, patients with an inverted-J-shaped required higher PEEPs to minimize dP_aw_ and presented higher BMI and lower initial PaO_2_/FiO_2_ ratio. In the only patient with this profile on EIT, PEEP decreased collapsed areas without increasing hyperdistention up to 20 cmH_2_O. The interpretation of the PEEP with respiratory system mechanics or with the amount of recruitment and overdistension on EIT seems to give the same information.

At least one-third of patients were obese in C-ARDS different cohorts [3, 7, 8], even though the effect of obesity on respiratory mechanics is well known, a relationship between BMI and compliance has not been described as an explanation, at least in part, for the COVID-19 different phenotypes. Obesity reduce Crs with the major contribution coming from the lung and not the chest wall [17] in spite of no significant association between compliance and BMI has been detected in a large cohort study of C-ARDS [18]. Mezidi et al. comparing a group of obese vs non-obese in C-ARDS patients monitoring esophageal pressure in a decremental PEEP trial demonstrated a significant difference in PEEP level for the same transpulmonary driving pressure (∆*P*_L_) and dP_aw_ [19]. ∆*P*_L_ also did not enhance significant information concerning the prediction of outcome in ARDS patients compared to dP_aw_ itself [20].

### Limitations

The observational nature of this study is its major limitation, and although data were acquired prospectively, they were interrogated retrospectively. The heavy workload upon COVID-19 pandemic made impossible to perform a clinical trial comparing clinical outcomes considering the observed profiles. The small proportion of patients investigated with EIT did not allow an appropriate comparison between the two methods, but data suggest a similar result to obtain the best PEEP for protective ventilation with a much simpler bedside procedure.

## Conclusion

The dP_aw_ vs PEEP curve is a feasible method and provides individualized information. A range of compliance and PEEPmin_dP_aw_ was observed in all 3 groups and its interpretation suggested that just in a minority of C-ARDS patients, higher PEEP improves compliance, and even in these cases, it appears that obesity, together with disease severity, determines this behavior. The overall influence of personalizing PEEP on clinical outcomes remains to be determined.

## Data Availability

The datasets used and/or analysed during the current study are available from the corresponding author on reasonable request.

## Abbreviations

ARDS: Acute Respiratory Distress Syndrome
AUC: Area under the curve
BMI: Body mass index
C-ARDS: ARDS from COVID-19
COVID-19: Corona Virus Disease-19
Crs: Respiratory system compliance
∆*P*_L_: Transpulmonary driving pressure
dPaw: Airway driving pressure
EIT: Electrical impedance tomography
FiO_2_: Fraction of inspired oxygen
IBW: Ideal body weight
ICU: Intensive care unit
MV: Mechanical ventilation
PEEP: Positive end-expiratory pressure
PEEP_EIT_: EIT optimal PEEP
PEEPmin_dP_aw_: The lowest PEEP that minimized dP_aw_
PaO_2_/FiO_2_: Partial pressure of arterial oxygen and fraction of inspired oxygen ratio
*V*_t_: Tidal volume
SOFA: Sequential organ failure assessment score
∆*Z*: Regional variation in impedance
ΔdPlow: Driving pressure at lowest PEEP
ΔdPhigh: Driving pressure at highest PEEP
